# Coverage and associated factors of vitamin-A supplementation among children aged 6-59 months in Gondar City, Northwest Ethiopia, 2022: a community-based cross-sectional study

**DOI:** 10.11604/pamj.2024.49.43.44537

**Published:** 2024-10-18

**Authors:** Kalkidan Berhane Tsegaye, Abel Sinshaw Assem, Destaye Shiferaw Alemu, Getenet Shumet Birhan, Biruk Lelisa Eticha

**Affiliations:** 1 Department of Optometry, College of Medicine and Health Sciences, Comprehensive Specialized Hospital, University of Gondar, Gondar, Ethiopia,; 2 Department of Clinical Epidemiology, Institute of Public Health, College of Medicine and Health Sciences, Comprehensive Specialized Hospital, University of Gondar, Gondar, Ethiopia

**Keywords:** Vitamin A deficiency, vitamin A, children

## Abstract

**Introduction:**

vitamin A is a nutrient required for normal visual system function, growth, and development. Periodic vitamin A supplementation is a cost-effective strategy for preventing vitamin A deficiency in children. This study aimed to assess the coverage and associated factors of vitamin A supplementation among children aged 6-59 months in Gondar City, Northwest Ethiopia 2022.

**Methods:**

a community-based cross-sectional study with a multistage random sampling technique was done on 587 mothers with 6-59-month-old children from October 20 to November 10, 2022, in Gondar City. The descriptive statistics are summarized by frequency, percent, and summary statistics. Binary logistic regression was performed, and variables with a P-value <0.05 were considered significantly associated.

**Results:**

the vitamin A supplementation coverage was 34.4% (95% CI (30.3% - 38.3%)). Children aged 6-13 months [AOR=9.50, 95% CI; (4.59-9.66)], 14-27 months [95% CI; (3.07-12.03)], mothers who had an education level of certificate or above [AOR=3.79, 95% CI; (1.45-9.90)], mothers who learned in secondary schools [AOR=3.29, 95% CI; (1.28-8.45)], mothers who had four or more antenatal care visits [AOR=4.32 (95% CI: (1.54-11.97) and mothers' good knowledge towards vitamin A [AOR: 2.20 (95% CI: 1.60-4.10)] showed a statistically significant association.

**Conclusion:**

the coverage of vitamin A supplementation exceeded the 70% UNISAFE threshold. A younger child's age, maternal education level, more than 4 antenatal visits, and good knowledge of vitamin A were significant factors. Extended and more integrated immunization programs with robust health education regarding vitamin A supplementation would play a prodigious role in getting higher coverage.

## Introduction

Vitamin A is one of the micronutrients required by the human body to function normally. In humans, it plays a variety of roles, from the formation of visual pigments to the regulation of growth hormone production [1]. Inadequate intake of vitamin A Deficiency (VAD) results in an impaired immune system, destabilized cellular integrity with severe manifestations of infection, and ocular manifestations including night blindness, corneal and conjunctival xerosis, and corneal ulcers and necrosis that lead to visual impairment and blindness [2-4]. VAD is a significant public health problem in developing countries [1,5]. Because of the higher requirement per body weight and higher incidence of infectious diseases, children under five years of age are the most vulnerable group to VAD [6,7]. An estimated 254 million children are vitamin A deficient which is attributable to 0.8 million deaths in children worldwide [8]. Globally, 1.5 million children are blind, 5 million are affected by night blindness, and about 350,000 people are going to be blind every year because of VAD. This makes VAD one of the major causes of preventable childhood visual impairment [9-12]. This fact eventually limits development, educational performance, and social and employment prospects [10].

Dietary diversification, vitamin A fortification with food, and vitamin A Supplementation (VAS) are the ways of food and medicine-based approaches used to deal with VAD and its burdens [2]. Supplementing with vitamin A is one of the most cost-effective strategies that reduce child mortality, general health-related morbidities, and ocular morbidities such as blinding corneal diseases; measles and xerophthalmia [13-16]. The World Health Organization (WHO) strongly recommends high-dose VAS for infants and children aged 6 to 59 months in settings where VAD is a public health problem like countries where the Under-Five Mortality Rate (U5MR) exceeds 70 deaths per 1000 live births. The guideline suggests administering 100,000 IU of VAS to 12-month-old children once and 200,000 IU twice for 12 to 59-month-old children [17].

However the global VAS coverage remains unsatisfactory, United Nations International Children's Emergency Fund (UNICEF) and WHO have been fighting VAD by using VAS as a major weaponry [2,13,14]. Providing the recommended dose of VAS for 80% or more of preschool children has been considered one of the successful interventions [18]. Ethiopia has been conducting VAS programs since 1995 in collaboration with UNICEF using the expanded program on immunization and then transformed the programme as a primary child survival strategy to supplement vitamin A to targeted children through outreach services biannually [10,19].

The range of VAS coverage reported from different regions in the globe so far was confirmed to be extended from 6.0% to 87.8% [20-24]. Whereas nearly half of the preschool children in sub-Saharan Africa did not get the supplement [25]. Factors associated with VAS coverage observed so far include mothers' age [26], mothers' job [27], family monthly income [26,28,29], maternal knowledge towards vitamin A [19,30,31], child age [19,31-33], maternal education level [28,32,34,35], hospital delivery [27], frequency of Antenatal Care (ANC) visit [22,26,28,29,36], and Postnatal Care (PNC) visit [28].

In developing countries such as Ethiopia, where the core of the health system is prevention, it is essential to study VAS coverage and associated factors. This may help to evaluate food and nutritional policies by comparing with WHO guidelines and can be used as additional information for the Ethiopian Ministry of Health's Health Sector Transformation Plan Two (HSTP II), which aims to strengthen and scale up VAS to children aged 6-59 months by the end of 2024 [18]. As revealed above, a wide variety of figures regarding VAS coverage and associated factors has been reported worldwide while quite limited evidence exists in the study area and Ethiopia at large. In order to support the endeavor to make informed decision-making regarding VAS in the world; especially in developing world, this study was conducted to assess the current VAS coverage and associated factors among children aged 6-59 months in Gondar City.

## Methods

**Study design:** a community-based cross-sectional study was conducted.

**Study period and area:** the study was conducted in Gondar City from October 20^th^ to November 10^th^ 2022. Gondar City is located in the North Gondar zone 748 km from the capital city of Ethiopia, Addis Ababa. It is the capital of the central Gondar administrator zone in the Amhara region, with an estimated total population of 395,138 and approximately 23,929 mothers of children under five years old [37]. Gondar is divided into 12 sub-cities that consist of 22 *kebeles* (the smallest administrative unit) hosting approximately 53,725 households. The city has eight public health centers, 14 health posts, 32 private clinics, and one referral hospital that provides delivery, ANC, Postnatal Care (PNC), and VAS vaccination services [38].

**Population:** the source population was mothers of children aged 6-59 months in Gondar City, and the study population was mothers of children aged 6-59 months living in the selected *kebeles* in Gondar City.

**Inclusion and exclusion criteria:** all mothers with children aged 6-59 months living in Gondar City for at least 6 months were included in the study, whereas mothers who were seriously ill and had difficulty communicating were excluded from the study.

**Variables of study:** the dependent variable was coverage of VAS.

### The independent variables

***Socio-demographic variables:*** they were childs age, child sex, maternal age, maternal education, paternal education, maternal occupation, number of children, and family monthly income.

***Clinical factors:*** they involved variables such as place of delivery, number of ANC visits, PNC checkup, time to reach health facility, previous history of under 5 years child death, and personal perception factors, including knowledge towards vitamin A and source of information.

### Operational definition

***Knowledge towards vitamin A:*** a respondent who correctly answered 6 (> 60%) and above questions among 10 Knowledge-related questionnaires on vitamin A was considered to have good knowledge, and those who scored <60% of the questions on vitamin A were considered poor knowledge [18,19,39].

***The vitamin A supplementation status:*** it was determined when a child received the proper age-specific dose of VAS (according to the WHO recommendation) (for children, 6-12 months old, once a year, and for 12-59-month-old children, twice a year) [14].

### Sample size and sampling techniques

***Sample size for the first objective:*** the sample size was determined using the single population proportion formula based on the following assumptions: level of significance (α): 5% (with a confidence level of 95%), marginal error: 5%, and P: 0.58 (58% of vitamin A supplementation coverage was taken from the Mini-Demographic and Health Survey from the Amhara region in 2019 [40]. The Z value was 1.96 (n: sample size, P: proportion of coverage, d: marginal error).


$$\text{n}=\frac{{\left(Z_{a/2}\right)}^{2}*P\left(1-P\right)}{d^{2}}$$

n= [(1.96)^2^x0.58(0.42)]/[0.05]^2^, n=374, total sample

***Sample size for the second objective:*** maternal knowledge of the VAS was significantly associated with VAS in different studies [19]. Sample size calculation was based on outcome variables and associated factors are presented (Table 1). Therefore, the largest sample size was 374. By using the design effect, 1.5= 56. Considering a 10% nonresponse rate, the final sample size was 617.

**Table 1:** sample size calculation based on outcome variables and associated factors

Variable	VAS practice		Total	COR (95%CI)	Sample size
	Good (%)	Poor (%)			
**Knowledge**					
Poor knowledge	151 (29.3)	364 (70.7)	515	1.00	
Good knowledge	90 (60)	60 (40)	150	3.6 (2.5–5.3)	160

Note:- VAS: Vitamin A Supplementation

**Figure 1:**
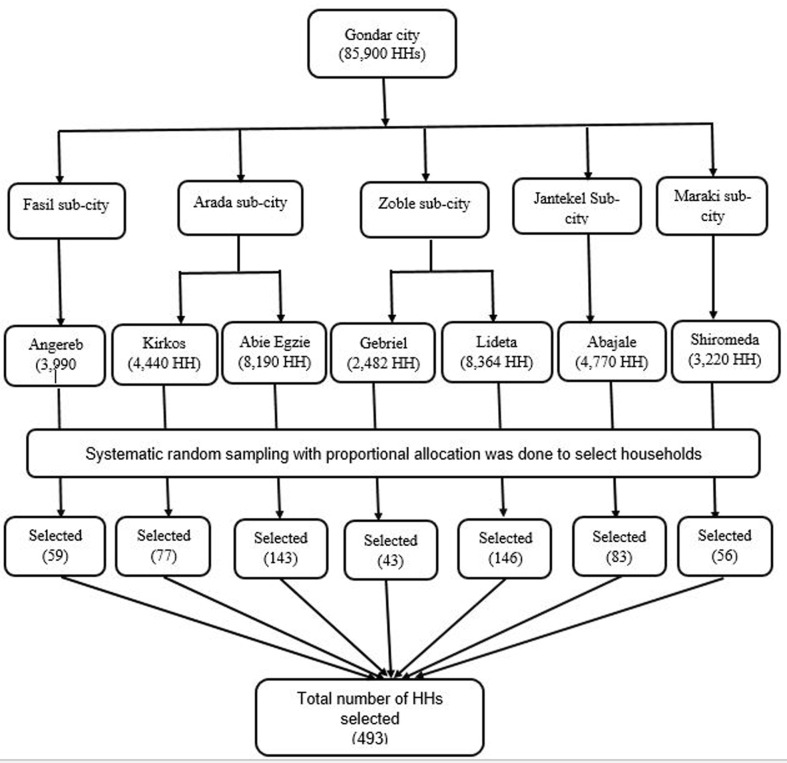
sampling technique summary used to select study subjects

***Sampling techniques and procedures:*** a multistage sampling technique using two stages of sampling processes was used. Once the list of the total *kebeles* in Gondar City was obtained from the local administration office, 30% of the *kebeles* were selected through a simple random sampling method. An estimated 35,456 households are found in these selected *kebeles* (Figure 1). Considering the selected *kebeles'* population, the minimum adequate sample size calculated was proportionally allocated to the selected *kebeles*. The sampling fraction (K=58) was determined by taking the ratio of households in the respective *kebele* to the sample size selected in each *kebele*. (i.e. k= Ni/ni, where Ni= total population in each selected *kebele* and ni= sample size taken from each selected *kebele*) The first sample unit was randomly selected, and every 58^th^ house was included in the study. When there was more than one eligible study subject in the household, only one child was included in the study via the lottery method [19]. In a situation where the eligible study subject was absent at the selected house, an adjacent house was substituted.

**Data collection tool, personnel, and procedure:** data were collected using a pretested and structured questionnaire that included information about sociodemographic characteristics, health service-related information, and mothers' knowledge and practices related to VAS. The questionnaire was prepared by reviewing related literature [13,18,41]. The questionnaire was developed in English, translated to Amharic, and subsequently translated back to English. The Amharic version of the structured questionnaire was used to collect the data.

**Data quality control:** the Amharic translated version of the questionnaire was pretested in the Bahir Dar on 21 mothers to test its interpretability and clarity. The reliability of the questionnaire was assessed using Cronbach's alpha. The questions used to assess the knowledge of the mothers were tested, and the value was 0.746. The questionnaire was translated from English to Amharic and then back to English to ensure consistency. There was training for data collectors for one day on the data collection procedures, including how to select the eligible subject and how to interview. The principal investigator and supervisor checked the collected data daily for completeness, accuracy, and clarity.

**Data processing and analysis:** the coded data were entered into Epi-info version 7 and exported to SPSS version 25 for analysis. Frequencies and cross-tabulations were used for descriptive analysis. The data are presented in tables and graphs. An adjusted odds ratio with a 95% confidence interval was used to measure the strength of the association between the outcome and the explanatory variables. The associations were assessed by binary logistic regression. Model fitness was checked using the Hosmer and Lemeshow goodness of fit test (sig= 0.862). Bivariable logistic regressions of variables with a P value < 0.2 were entered into the multivariable analysis, and those with a P value < 0.05 were considered to be statistically significant.

**Ethical approval:** ethical clearance with a reference number of SOM/1556/2022 was obtained from the University of Gondar, College of Medicine and Health Sciences, School of Medicine Ethical Review Committee according to the declaration of Helsinki. Before data collection, verbal informed consent was obtained from each respondent. In this regard, their full right to withdraw or refuse to participate in the study was respected. Respondents' data was collected without an identifier.

## Results

**Socio-demographic characteristics of the study participants:** a total of 587 participants completed the study with a response rate of 95.1%. The median ages of the mothers and the children were 30 years and 27 months with Interquartile Range (IQR) of 26-35 years and 14-42 months, respectively. Approximately 80.9% of the mothers were married. On the contrary 8.0% could not read or write. Almost half of the study subjects were housewives (Table 2).

**Table 2:** socio-demographic characteristics of mothers of children aged 6-59 months in Gondar City, Northwest Ethiopia, 2022 (n=587)

Variables	Frequency	Percent
**Age of mothers in years**		
15-24	107	18.2%
25-34	309	52.6%
35-42	171	29.2%
**Age of child in months**		
6-23	258	44.0%
24-59	329	56.0%
**Child sex**		
Male	280	82.4%
Female	307	17.6%
**Mother’s marital status**		
Married	475	80.9%
Divorced	56	9.5%
Single	41	7.0%
Widowed	15	2.6%
**Mothers’ educational status**		
Cannot read and write	47	8.0%
Can read and write	94	16.0%
Elementary	95	16.2%
Secondary	156	26.6%
Certificate and above	195	33.2%
**Mother’s occupation**		
Housewife	291	49.6%
Government worker	107	18.2%
Private business	169	28.8%
Daily laborer	20	3.4%
**Father’s educational status**		
Cannot read and write	20	3.4%
Can read and write	36	6.1%
Elementary	70	11.9%
Secondary	139	23.7%
Certificate and above	204	34.8%
Unknown	118	20.1%
**Father’s occupation**		
Daily labor	56	9.6%
Private business	125	21.3%
Government employee	154	26.2%
Private employee	134	22.8%
Unknown	118	20.1%
**Number of children in the household**		
1	212	36.1%
2	148	25.2%
≥ 3	227	38.7%

**Clinical characteristics of the study participants:** more than half of the mothers (53.5%) delivered their child in the hospital. Approximately 546 (93.0%) of the mothers had received ANC service during their pregnancy period. Among those, two-thirds (64.6%) of them visited ANC unit more than four times. On the other hand, one-third (34.6%) of the study subjects had no PNC visit (Table 3).

**Table 3:** clinical characteristics of mothers of 6- to 59-month-old children in Gondar City, Northwest Ethiopia, 2022 (n=587)

Variables	Frequency	Percent
**Place of delivery**		
Hospital	314	53.5%
Health center	146	24.9%
Private clinic	59	10.0%
Home	68	11.6%
**ANC visit**		
No	41	7.0%
Yes	546	93.0%
**Number of ANC visit**		
None	41	7.0%
1-3 visits	167	28.4%
≥ 4 visits	379	64.6%
PNC visit		
No	203	34.6%
Yes	384	65.4%
**History of death of > 5 years child**		
No	489	83.3%
Yes	98	16.7%
**Distance of health institution**		
< 30 minutes	456	77.6%
≥ 30 minutes	131	22.4%

Note:- ANC: Antenatal Care, PNC: Postnatal Care

**Knowledge of mothers towards vitamin A:** of the 587 mothers, 249 (42.4%) had good knowledge of vitamin A. Approximately 66.3% (389) had heard about vitamin A. Four hundred and one mothers had seen the VAS capsule. Additionally, 177 mothers were informed of the correct month at which the VAS should be started. However, only 11.1% of them knew how frequently VAS should be given. Among the 219 mothers who knew about the effect of VAD, 88 knew that VAD can cause night blindness, and only 40 mothers reported permanent loss of sight as an effect of VAD. Regarding their source of information, more than 2/3 (318) of mothers found the information from health professionals, while 209 mothers found this information from health extension workers. However, 60 of them had other information sources, such as mass media.

**Figure 2:**
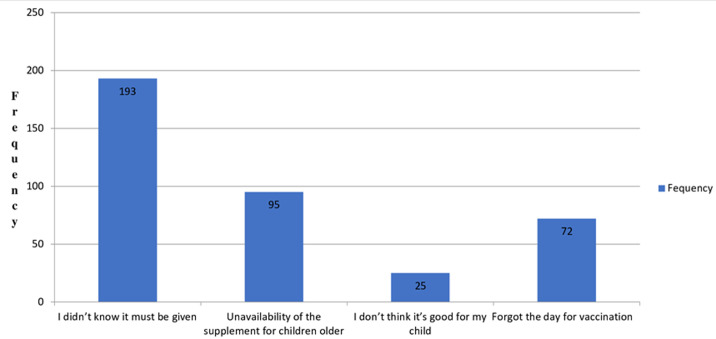
reasons of mothers for not giving vitamin A supplement for their child

**Coverage of the VAS score:** among the 587 mothers, 445 (75.8%) reported that their child had received VAS. Among them, 403 received routine immunization, while only 21 mothers were able to provide the supplement themselves. Of the participants, 272 children received VAS twice or more, while 38.8% of them received VAS only once. In this study, the VAS coverage is found to be 34.4% (95% CI (30.3% - 38.3%)). Among the remaining 385 children, 95 did not receive VAS as recommended because of the unavailability of the supplement in health centers or hospitals for children older than 15 months (Figure 2).

**Factors associated with mothers' practices on the VAS:** according to the bivariable analysis, maternal age, child age, child sex, mother educational status, maternal occupation, number of children in the household, monthly income, place of delivery, number of ANC visits, PNC visit, history of under 5-year mortality in family, source of information and knowledge were factors associated with good practice according to the VAS. However, according to the multivariate logistic regression analysis, child age, maternal education status, number of ANC visits and knowledge were factors significantly associated with supplementation of VAS. The odds of receiving VAS were ten times more likely among children aged 6-14 months (AOR=9.50, 95% CI=4.59-9.66) and six times greater for those aged 14-27 months (AOR= 6.08, 95% CI=3.07-12.03) than for those aged 43-59 months.

Children with mothers who had an education level of certificate and above were nearly four times more likely to receive VAS [AOR=3.79, 95% CI; (1.45-9.90)] than children with mothers who could not read or write. Additionally, mothers who learned in secondary school had a threefold greater chance of receiving VAS [AOR= 3.29, 95% CI= 1.28-8.45]. Mothers who had four or more ANC visits [AOR = 4.32 (95% CI: (1.54-11.97)] were four times more likely to receive vitamin A capsules for their children than mothers who had no ANC visits. Children whose mothers had good knowledge of vitamin A had received vitamin A capsules were two times more likely than children whose mothers had poor knowledge [AOR: 2.20 (95% CI: 1.60-4.10)] (Table 4).

**Table 4:** factors associated with VAS coverage among children aged 6-59 months in Gondar City, Northwest Ethiopia, 2022

Variable	Vitamin A supplementation		COR (95%CI)	AOR (95%CI)
	Yes	No		
**Age of mothers in years**				
15-24	46	61	1.00	1.00
25-34	110	199	0.73(0.46-1.14)	0.63(0.33-1.21)
35-47	46	125	0.48(0.29-0.81)	1.25(0.81-1.93)
**Age of child in a month**				
6 -23	139	119	4.93(3.05-8.9)	6.54(4.02-10.63)***
24-59	63	266	1.00	1.00
**Child sex**				
Male	91	189	1.00	1.00
Female	111	196	1.17(0.83-1.65)	1.25(0.81-1.92)
**Mother's educational status**				
Cannot read and write	10	37	1.00	1.00
Can read and write	10	84	0.44(0.16-1.14)	0.77(0.25-2.41)
Elementary	23	72	1.18(0.50-2.74)	1.77(0.63-4.95)
Secondary	64	92	2.57(1.19-5.54)	3.29(1.28-8.45)*
Certificate and above	95	100	3.51(1.65-7.46)	3.79(1.45-9.90)**
**Average family monthly income in ETB**				
600-1000	61	173	1.00	1.00
1001-4000	69	77	1.80(1.14-2.86)	0.91(0.49-1.69)
4001-7000	50	72	1.96(1.23-3.13)	1.15(0.58-2.51)
7001-17000	42	63	1.89(1.16-3.07)	0.71(0.34-1.47)
**Number of children**				
1	76	136	1.55(1.03-2.33)	0.75(0.39-1.46)
2	66	82	2.24(1.44-3.47)	1.25(0.70-2.24)
≥ 3	60	167	1.00	1.00
**Place of delivery**				
Hospital	101	213	1.13(0.64-2.01)	0.45(0.18-1.08)
Health center	48	98	1.17(0.62-2.19)	0.47(0.19-1.16)
Private clinic	33	26	3.04(1.46-6.33)	1.02(0.36-2.89)
At home	20	48	1.00	1.00
**Number of ANC visit**				
None	8	33	1.00	1.00
1-3 visits	52	115	1.86(0.80-4.31)	2.34(0.81-6.76)
≥ 4 visits	142	237	2.47(1.11-5.50)	4.30(1.54-11.97)**
**PNC visit**				
Yes	144	240	1.50(1.03-2.16)	1.02(0.61-1.70)
No	50	145	1.00	1.00
**History of death of > 5 years child**				
Yes	22	76	1.00	1.00
No	180	309	0.49(0.29-0.82)	1.13(0.60-2.11)
**Knowledge**				
Good	133	116	4.47(3.10-6.42)	2.20(1.60-4.10)***
Poor	69	269	1.00	1.00
**Source of information**				
Health workers	71	247	0.17(0.10-0.32)	0.15(0.62-4.13)
Health extensions	94	115	0.50(0.28-0.91)	0.13(0.17-1.79)
Mass media	37	23	1.00	1.00

Note:- *: P value < 0.05, **: P value < 0.01, ***: P value < 0.001, ETB: Ethiopian Birr, ANC: Antenatal Care, PNC: Postnatal Care.

## Discussion

This population-based cross-sectional study investigated the practices of mothers of 6-59-month-old children on VAS and the associated factors in Gondar City, Northwest Ethiopia. In the present study, the proportion of VAS coverage was 34.4% (95% CI (30.3% - 38.3%)). The age of the child, maternal educational level, number of ANC visits, and maternal knowledge of vitamin A were factors that were significantly associated with coverage of VAS. The proportion of coverage of VAS (34.4%) found in this study is in line with that found in Ethiopia (36.2%) [19].

The proportion of coverage (34%) found in this study is less than that found in Ethiopia (44.4%) and (58.0%) [28,29], Kenya (52.0%) [21], Nigeria (41.6%) [42], Tanzania (53.1%) [27], India (52.2%) and (87.8%) [22,23] and Bangladesh (63.5%) [20]. This difference might be due to the difference in the study setting. The study in Kenya was hospital-based, while this study was population-based. This great discrepancy in the results from India and Bangladesh might be due to differences in operational definitions. The authors considered coverage for children who had received VAS at least once, while this study included coverage for children who received VAS every 6 months.

However, this study's finding is higher than that reported in India (6.2%) [24]. The reason behind this difference might be that the subjects of the Indian study were mothers who lived in urban resettlement areas. The majority of them were migrating workers. This displaced population has a low socioeconomic status and unstable life. This socioeconomic and demographic difference may have led to these lower results [33].

Mothers' knowledge of the VAS is an associated factor with VAS. Being the child of a mother having good knowledge of vitamin A gave two times more odds of taking VAS than being born from a mother having poor knowledge of vitamin A. These findings are supported by other studies from Ethiopia [19,28], Libya [30] and Ghana [31]. This might be because mothers with good knowledge about vitamin A can be conscious of morbidity, mortality, and blindness due to VAD and the use of VAS to fight against this deficiency [43]. This would have influenced the mothers to be attached to vitamin A supplementation.

According to the present study, the younger age of the child was positively associated with VAS coverage. Children aged 6-14 months were 10 times more likely to receive VAS than children aged 42-59 months. These findings were also reported in studies done in Ethiopia [19], Ghana [31] and India [24,33]. This could be because of the routine vaccine program administered for up to 15 months in Ethiopia and 24 months in India [19]. However, younger age has been shown to be negatively associated with VAS in studies done in Bangladesh and twenty-eight sub-Saharan countries, where it was reported that children aged 6-11 months had a lower likelihood of receiving vitamin A [32,33]. This may be because of the underestimation of study participants closer to the lower limit of the respective age brackets. For instance, the first dose of vitamin A may not yet be given to a 6-month-old infant, yet it is considered to not be supplemented [44].

In this study, maternal education was another factor that was positively associated with VAS coverage. Children with mothers who had certificates and above were three times more likely to receive VAS than children with mothers who had no formal education. These findings are supported by studies performed in India [45], Bangladesh [32], Indonesia [34] and Cambodia [35]. This finding may be explained by formal education increasing health and nutritional awareness, which leads to increased practice [43,46]. On the other hand, a study in Ethiopia [47] found no association between the mother's education level and the child's VAS. This result may be explained by the fact that the study was performed with secondary data from the Ethiopian Demographic Health Survey (EDHS). The survey was held after a vaccination campaign, which made the mothers aware of the VAS. As such, their educational status may be less relevant than whether they were reached by campaigns. Unexpectedly, in another study done in Ethiopia, compared with illiterate mothers, those in the Humbo District reported a 47% reduction in the likelihood of receiving VAS for children born to literate mothers [18]. This negative association was also found in Bangladesh [48]. This might be due to an error that emanates from chance.

The number of ANC visits was also found to be significantly associated with VAS recipients in this study. Children of mothers who had four or more ANC visits were four times more likely to receive vitamin A capsules than mothers who had no ANC visits. These findings were supported by a study in Nigeria [36], Tanzania [27], and by national studies [22,26,29,49]. This could be explained by the fact that while receiving ANC service during pregnancy, health professionals encourage, educate, and counsel mothers about the benefits of vitamin A supplementation and the consequences of VAD. This information about VAS and immunizations can increase the chance of receiving a vitamin A capsule for their children [22,23].

**Limitations of the study:** the VAS information was based on the report of the mothers this may cause recall bias due to interactions with other vaccines, such as polio, and the study failed to assess health facility-related factors.

## Conclusion

In conclusion, the proportion of VAS coverage in this study was very low compared with the WHO recommendation of 80% and the Health Sector Transformation Plan I (HSTP I) target of 95%. Age of the child, maternal educational status, knowledge of mothers on the VAS, and time taken to reach a health facility were factors associated with Vitamin A coverage. Based on our findings, health promotion and education activities aimed at increasing mothers' knowledge of Vitamin A and VAS are recommended.

### What is known about this topic

- Prior studies have documented a high prevalence of vitamin A deficiency among children in Ethiopia, for instance, the Ethiopian Demographic and Health Survey (EDHS) indicated that a significant proportion of children under five years old experienced vitamin A deficiency, which poses risks for vision impairment and increased susceptibility to infections;
- Various interventions are being implemented to address vitamin A deficiency, including supplementation programs and fortification efforts; however, coverage and impact have varied across regions;
- Studies have shown that while supplementation programs have improved vitamin A status in some areas, challenges remain in achieving comprehensive coverage and reaching all vulnerable populations.

### What this study adds

- This study was able to investigate the full spectrum uptake of vitamin A supplement recommended, to come up with vital data regarding the uptake related status;
- This information can guide more targeted and effective intervention strategies by bringing value and adding information to policy makers used to figure out region related variation;
- The study uncovers specific logistical and socio-cultural barriers affecting the distribution and uptake of vitamin A supplements, understanding these barriers can help in designing strategies to improve program efficiency and outreach.
